# Supplementary material for: Enhancement of 3D Printability by FDM and Electrical Conductivity of PLA/MWCNT Filaments Using Lignin as Bio-Dispersant
Source: Polymers (Basel). 2023 Feb 17;15(4):999. doi: 10.3390/polym15040999 (PMC9960198; doi:10.3390/polym15040999)
Supplement: Supplementary file 1 [file polymers-15-00999-s001.zip › polymers-2222430-supplementary.pdf]

Solvent optimization study

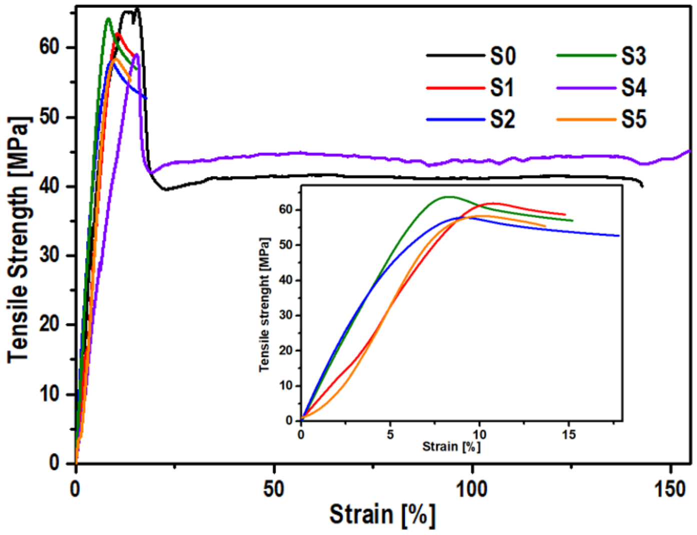

Figure S1.- Stress-strain curves obtained from tensile tests. Samples involved in the solvent optimization study. Inset image: plot amplification with the strain between 0 and 20% to see the differences.

Optimization of MWCNT content

w	η*[Pa s]					
[rad·s^{-1}]	PLA	P2C	P4C	P6C	P8C	P10C
0.1	1.08x10^2	5.63x10^4	3.17 x10^5	2.30x10^6	3.77 x10^6	4.69x10^6
1	1.51x10^2	5.94x10^3	3.60 x10^4	2.64x10^5	3.90x10^5	4.86x10^5
10	1.47x10^2	8.63x10^2	4.29 x10^3	2.91x10^4	4.36x10^4	5.49x10^4
100	7.48x10^1	2.37x10^2	7.07x10^2	3.28x10^3	5.01x10^3	6.67x10^3

Table S1.-Complex viscosity of several PLA/MWCNT composites with different amounts of nanofiller measured at different frequencies. PLA is an injected PLA sample for reference.

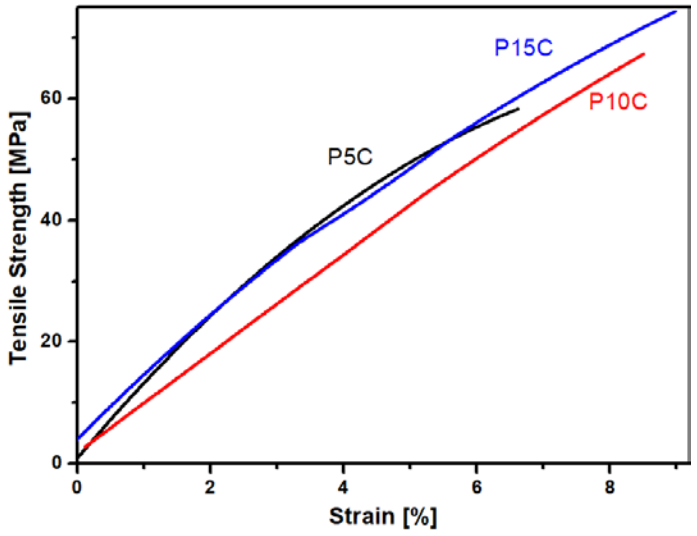

Figure S2.- Stress-strain curves obtained from representative samples of tensile tests of PLA/MWCNT composites without additives.

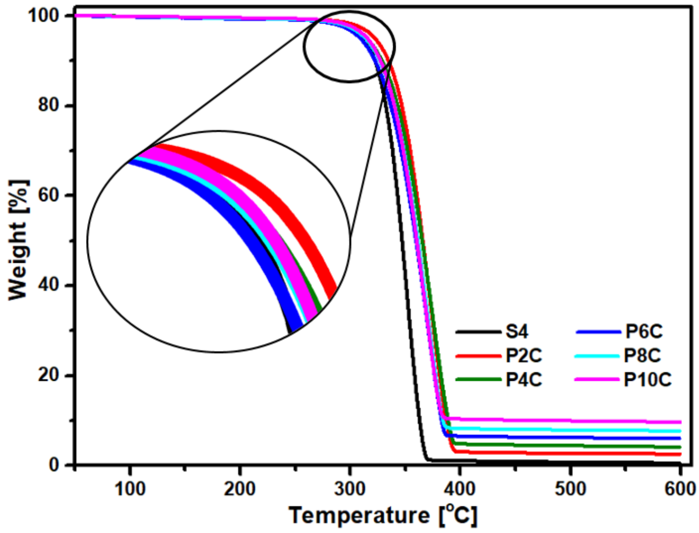

Figure S3-TGA thermograms of several PLA/MWCNT samples and S4 reference sample.

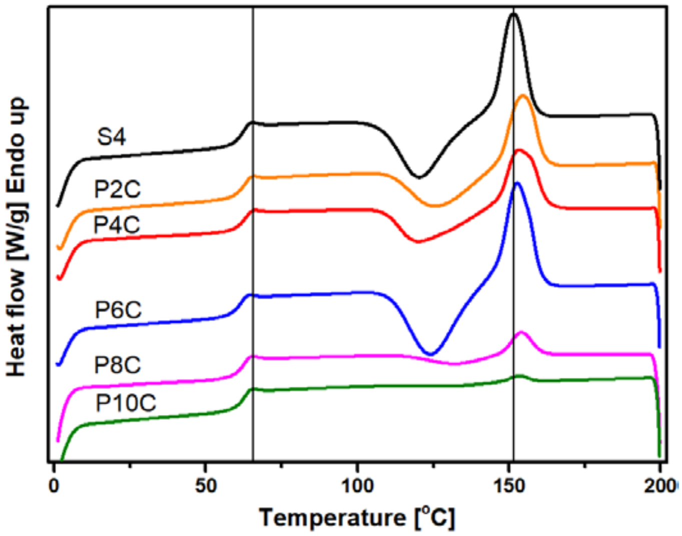

Figure S4.-DSC thermograms of MWCNT/PLA composites without PEG and S4 reference sample.

Sample	Tcc [°C]	ΔHcc [$J \cdot g_{PLA}^{-1}$]	Tm [°C]	ΔHm [$J \cdot g_{PLA}^{-1}$]	σ [$S \cdot cm^{-1}$]
S4	120.3	23.5	151.3	24.7	-
P2C	124.5	18.8	154.5	18.9	$(1\pm1)\cdot10^{-8}$
P4C	122.4	14.3	154.0	15.15	$(3.7\pm1.4)\cdot10^{-8}$
P4.5C	-	-	-	-	$(1.5\pm0.8)\cdot10^{-7}$
P5C	-	-	-	-	$(2.8\pm1.5)\cdot10^{-7}$
P5.5C	-	-	-	-	$(2.1\pm1.3)\cdot10^{-1}$
P6C	124.7	29.5	153.1	29.7	$(5.1\pm1.1)\cdot10^{-1}$
P8C	123.8	6.1	154.4	7.7	$(9.8\pm1.6)\cdot10^{-1}$
P10C	-	1.4	153.0	1.1	1.8 ± 0.4
P15C	-	-	-	-	4.0 ± 0.5

Table S2.-DSC and electrical conductivity data results of MWCNT/PLA composites without additives.

Optimization of plasticizer content

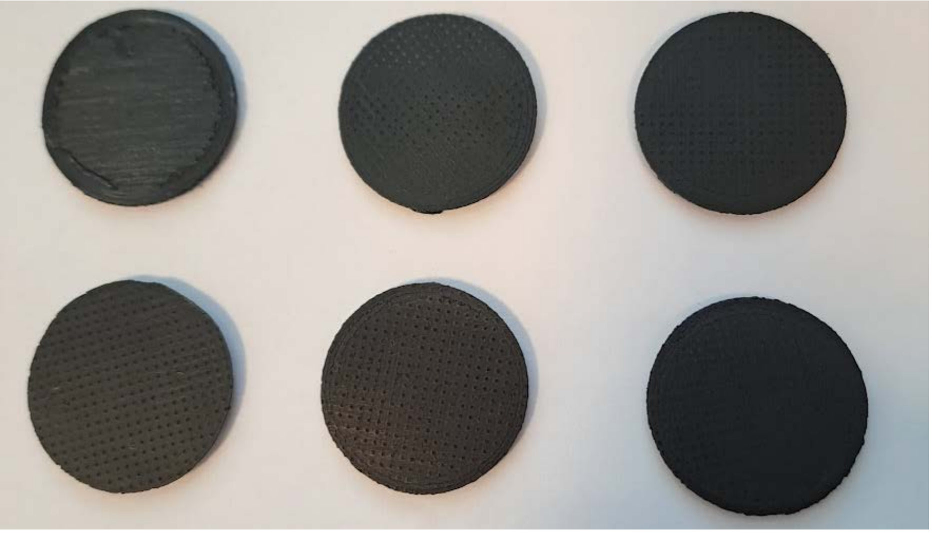

Figure S5-3D printed samples of PLA/MWCNT with (top) PEG and (botton) lignin as additive. From left to right, they possess 1-3 wt.% of additive..

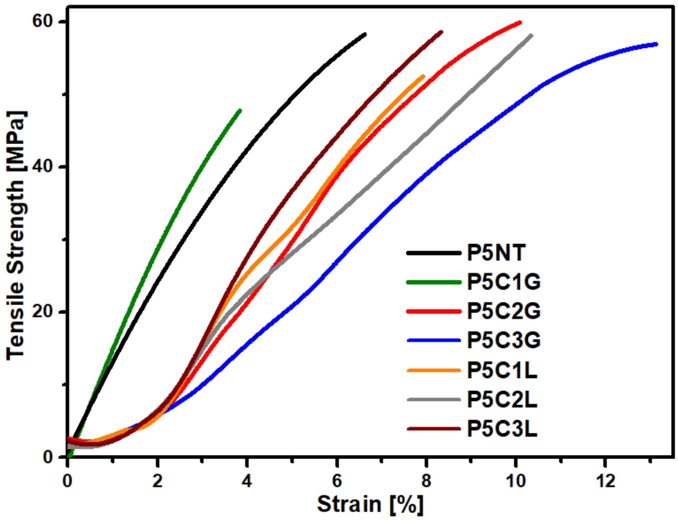

Figure S6-Stress-strain curves obtained from representative tensile tests of PLA/MWCNT/PEG composites with different amount of additives, A) lignin and B) PEG, and 5% of MWCNT.
